# Study on the factors influencing the prognosis after perianal abscess surgery

**DOI:** 10.1186/s12876-023-02959-1

**Published:** 2023-09-27

**Authors:** Shujiang Dong, Bingxue Chen, Jian Zhang

**Affiliations:** 1 Department of anus-intestines, Changzhou Second People’s Hospital Affiliated to Nanjing Medical University, No. 68 Gehu Middle Road, Wujin District, 213000, 213000 Changzhou, China; 2 Department of Medical Imaging, Changzhou Second People’s Hospital Affiliated to Nanjing Medical University, Changzhou, 213000 China

**Keywords:** Perianal abscess, Recurrence, Anal fistula, Influencing factors

## Abstract

**Objective:**

To study the influence of clinical characteristics and diagnosis and treatment methods of perianal abscess on postoperative recurrence or formation of anal fistula to provide a basis for selecting appropriate surgical and inspection methods for clinical treatment of perianal abscess in the future.

**Methods:**

The clinical data of 394 patients with perianal abscesses were collected, the influencing factors were investigated, and univariate analysis and multivariate logistic regression analysis were performed to further determine the risk factors affecting the prognosis of perianal abscess.

**Results:**

The results showed that the rate of preoperative blood routine results in the uncured group was higher (51.16%) than in the cured group (35.61%); the rate of high abscess space in the uncured group (23.26%) was higher than in the cured group (9.11%); the proportion of patients in the uncured group who underwent magnetic resonance imaging (MRI) before surgery (27.90%) was lower than in the cured group (45.30%); the proportion of patients in the uncured group who underwent simple drainage (51.16%) was higher than in the cured group (28.49%). The two groups had significant differences in perineal MRI examination, surgical method, preoperative blood routine, and abscess space (*p* = 0.030, *p* = 0.002, *p* = 0.047 and *p* = 0.010, respectively). Based on the results of univariate analysis and multivariate logistic regression analysis, the extent of the abscess cavity (OR = 2.544, 95%CI = 1.087–5.954, *p* = 0.031) and the surgical method (OR = 2.180, 95%CI = 1.091–4.357, *p* = 0.027) were independent influencing factors for postoperative recurrence of perianal abscess or anal fistula.

**Conclusion:**

Preoperative assessment of the abscess range and precise intraoperative methods to resolve the infection of the abscess glands in the internal mouth can effectively improve the cure rate.

**Established knowledge of the topic**.

1. Most perianal abscesses are caused by anal gland infection.
2. Surgical treatment is usually the first choice when clinically treating perianal abscesses.
3. Currently, the commonly used surgical treatments can be divided into three types: abscess incision and drainage, abscess incision and thread drawing and minimally invasive surgery.
4. Abscess incision and drainage or abscess incision and thread drawing are simple to operate but usually require a second operation.

**Study findings**.

1. Diabetes mellitus has no significant difference in the effect of postoperative abscess healing.
2. The range of the abscess space is an independent influencing factor for postoperative perianal abscess healing.
3. The operation method is an independent influencing factor for postoperative healing of perianal abscesses.

**How this study might affect research, practice or policy**.

1. Intraoperative thread-hanging radical drainage is more effective than simple incision and drainage to prevent postoperative recurrence and anal fistula formation.
2. The patient’s preoperative blood routine range can indicate the severity of inflammation and infection.
3. Imaging evaluation shall be perfected as much as possible before the operation, the internal mouth thoroughly explored during the operation and drainage performed.

## Introduction

The annual incidence of perianal abscesses in the UK is estimated to be between 14,000 and 20,000, with about 12,500 operations annually [1]. Most perianal abscesses are caused by anal gland infection and spread upward and downward, causing them to expand to the gaps around the anorectal canal, resulting in acute or chronic purulent inflammation [1–3]. In addition, perianal abscesses may also be caused by Crohn’s disease, sweat gland infection, human immunodeficiency virus, radiation therapy and skin infection [4–6]. The location of the abscess cavity is classified as perianal, ischioanal, intersphincteric and supralevator [7]. If it cannot be treated in time, it will usually cause the abscess to increase in volume or even rupture, so the pus cannot be discharged completely, leading to the recurrence of the perianal abscess [8]. In addition, abscesses may also cause systemic infections and spread rapidly, leading to tissue infections and gangrene. Therefore, timely treatment of perianal infections is essential [9–11].

Surgical treatment is usually the first choice for clinical treatment of perianal abscesses [12–14]. Currently, the commonly used surgical treatments can be divided into three types: abscess incision and drainage, abscess incision and thread drawing and minimally invasive surgery. Incision and drainage of the abscess usually involve an incision in the centre of the abscess to drain the pus. It is simple to operate and can effectively control the further development of perianal abscesses and protect the function of the anus to a certain extent. However, the rate of recurrence and sequelae such as anal fistula are high, and it usually requires a second operation, which has a longer course of disease [15–17]. Abscess incision and suture is a surgical method in which the internal mouth, the primary infected lesion, is found through a probe after cutting the abscess and draining the pus, and the rubber band is introduced. This treatment method can effectively prevent postoperative adverse reactions and avoid secondary operations and is commonly used in clinical practice. In addition, minimally invasive surgery is expected to achieve the greatest therapeutic effect through the smallest wounds and improve patients’ quality of life. However, current research on minimally invasive therapy is not comprehensive enough; here, we use abscess incision and drainage and abscess incision and thread drawing to treat patients.

Abscess complexity, lack of surgeon experience or patients with chronic systemic diseases often lead to a poor prognosis, most often the recurrence or formation of anal fistula within 1 year, so another operation is performed [18–21]. There are few studies on the status and incidence rate of perianal abscesses in China. Based on this, this study analysed the clinical data of patients with perianal abscesses undergoing surgical treatment in our hospital from January 2017 to October 2019 to explore the factors affecting prognosis after perianal abscess surgery.

## Materials and methods

### General information

**Fig. 1:**
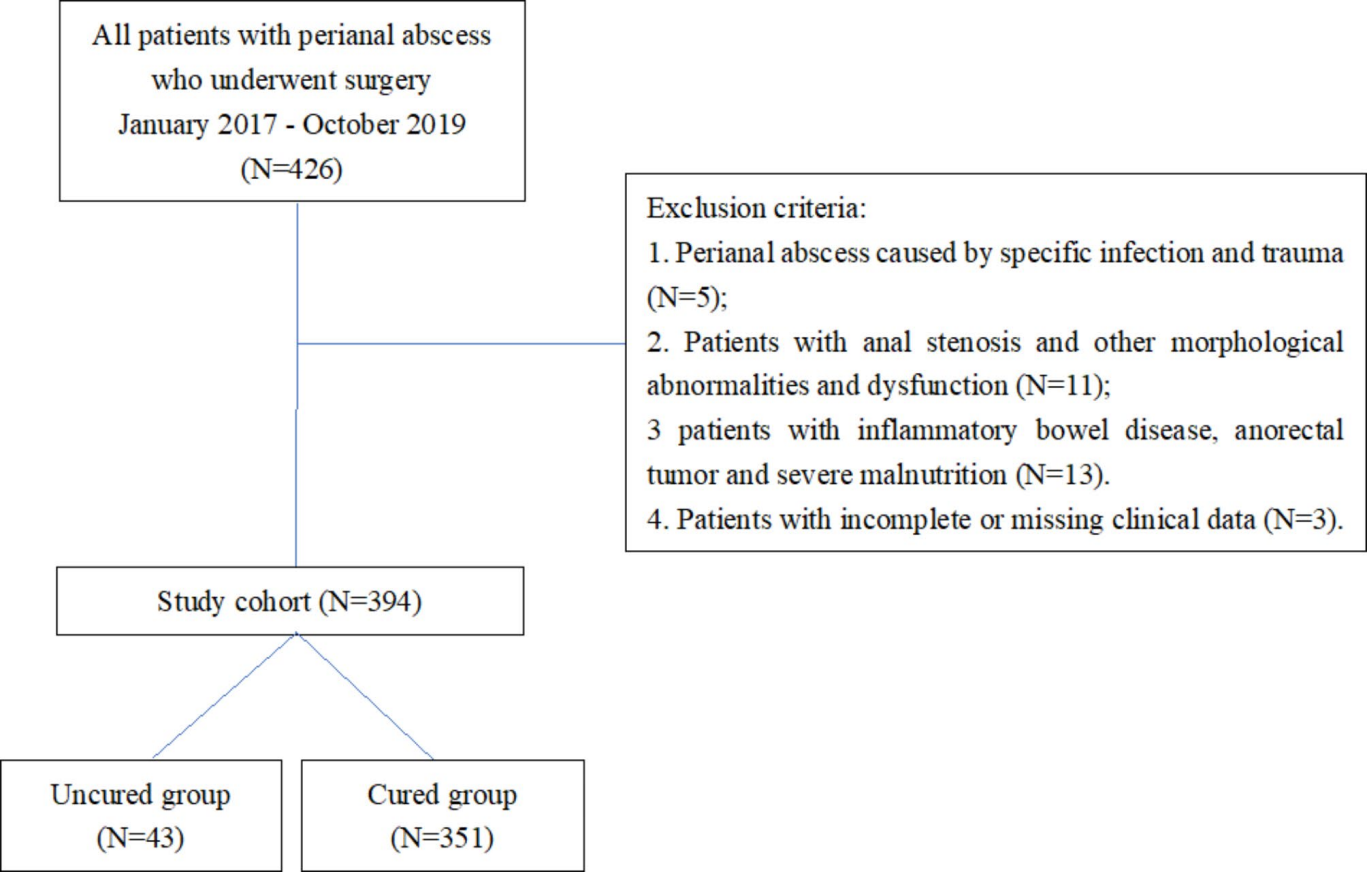
The flow chart of study design

This study is retrospective. The clinical data of patients diagnosed with perianal abscesses and undergoing surgical treatment admitted to our hospital from January 2017 to October 2019 were collected. Postoperative outpatient follow-up or telephone follow-up was maintained to assess recovery within 1 year. All patients were excluded from surgical contraindications and signed an informed consent form for surgical treatment before surgery. The study has been approved by the hospital’s medical ethics committee. See Fig. 1 for the flow diagram.

### Inclusion and exclusion criteria

Inclusion criteria: (1) Patients with perianal abscesses were confirmed, and age and sex were not limited; (2) Patients who underwent surgical treatment and underwent long-term outpatient follow-up dressing changes.

Exclusion criteria: (1) Perianal abscess caused by specific infection and trauma; (2) Patients with anal stenosis and other morphological abnormalities and dysfunction; (3) Patients with inflammatory bowel disease, anorectal tumour and severe malnutrition; (4) Patients with incomplete or missing clinical data.

Based on inclusion and exclusion criteria, 394 patients were finally enrolled in the study.

### Grouping

According to whether an anal fistula recurred or formed within 1 year after the operation, patients were divided into an uncured group and a cured group. Their clinical manifestations conformed to the ‘Clinical practice guideline for the management of anorectal abscess, fistula-in-ano, and rectovaginal fistula’ defined by the American Society of Colorectal Surgeons [22]. When the patient has any of the following conditions, they are classified in the uncured group: symptoms such as redness, pain, mass ulceration and pus perianal appear again; the perianal fistula or induration is clear, and there is usually repeated discharge; imaging examination (B-mode ultrasound, magnetic resonance imaging (MRI)) suggests the recurrence of perianal abscess or anal fistula formation. If the patient does not have the above-mentioned conditions during the follow-up period, they are classified in the cured group.

### Surgical methods

This study used two surgical methods performed by experienced chief physicians to investigate the impact of different surgical techniques on the postoperative prognosis. The first surgical method is abscess incision and drainage, which is performed under epidural anaesthesia or general anaesthesia. According to the preoperative digital anus examination or combined with MRI, an incision is made where the abscess is located or fluctuating the most. The abscess cavity drains the pus and removes the inflammation and necrotic tissue of the lesion. The incision is left open for drainage, or a tube is placed for flushing and drainage. The second surgical method is abscess incision and thread drawing, conducted under preoperative epidural anaesthesia or general anaesthesia. According to preoperative digital anus examination or combined with MRI, an incision is selected at the best drainage of the anal margin to remove pus and adequately drain the space of each abscess cavity to remove inflamed tissue. A vascular clamp is used to explore the internal orifice according to the suspicious anal gland infection suggested by MRI, and an elastic rubber band is used to drain the weak internal orifice with the combination of virtual and solid with thread hanging. Two kinds of antibiotics were routinely used for 5–7 days after the operation, and the dressing was changed daily.

### Wexner score

The Wexner score is an important indicator for evaluating anal function to investigate the recovery of patients with perianal abscesses, with a low score showing good patient recovery. The Wexner score was used to evaluate the anal function of the patients during the postoperative follow-up. The evaluation takes into account various factors, including the ability to control solids, liquids and gases, the need for sanitary pads and any changes in lifestyle. The total score is the sum of the scores, and the range is 0–20 points, with 0 points representing normal defecation and 20 points for complete incontinence.

### Observation indicators

Follow-up was conducted with patients who met the inclusion and exclusion criteria after surgery to determine the incidence of postoperative adverse reactions, add the patients’ Wexner scores and compare the two groups of clinical data, including age, gender and preoperative blood routine range. The data also included whether patients have combined diabetes, MRI examination of the perineum, the range of the abscess cavity and the operation method to study the relationship between them and the postoperative prognosis.

### Statistical analysis

The SPSS 22.0 software was used for statistical analysis. Measurement data are expressed as mean ± standard deviation, count data as (n), the comparison between groups is by χ^2^ test, and prognostic factors after perianal abscess surgery are analysed by multivariate logistic regression analysis. The difference was statistically significant with *p* < 0.05.

## Results and discussion

### The prognostic effect of perianal abscess after surgery

Among 394 patients, there were 329 males and 65 females, aged 11–80 years old, with an average age of 39.4 ± 12.3 years; 247 cases had normal blood routine examinations before operation (147 cases of abnormally high white blood cell count); 24 cases had diabetes mellitus; 171 cases had MRI examination before operation, and 223 cases did not undergo MRI examination; 352 cases had low perianal abscesses in the abscess space, and 42 cases had high perianal abscesses; 122 cases were operated by simple incision and drainage, and 272 cases had thread-attached drainage.

The wound recovered well after the operation, with occasional itching and dampness at the wound scar recorded as cured and adverse reactions such as recurrence or formation of anal fistula recorded as uncured. According to statistics, there were 351 cases (89.08%) in the cured group, 43 cases (10.92%) in the uncured group, of which 32 cases recurred or formed anal fistula within half a year, and 11 cases recurred within 6 − 12 months.

### Wexner score

The postoperative Wexner scores of 394 patients were statistically recorded. Among them were 363 patients with a score of 0, 16 patients with a score of 1, 11 patients with a score of 2, and 4 patients with a score of 3.

### Univariate analysis of the prognosis of perianal abscess after surgery

To investigate the factors that affect the prognosis of perianal abscess after surgery, the age, gender, preoperative blood routine, preoperative MRI, abscess gap, surgical method, and whether or not diabetes was combined in the two groups were analysed. The results are recorded in Table 1. The results showed no statistically significant difference in gender, age, and whether the two groups of patients with diabetes mellitus (p > 0.05).

**Table 1:** Single factor analysis of prognosis of perianal abscess after operation

Factor	Cure group (n = 351)	Uncured group (n = 43)	*χ* ^*2*^ */t*	*P*
Age (years)				
> 60	23 (6.55%)	3 (6.98%)	0.11	0.916
≤ 60	328 (93.44%)	40 (93.02%)		
**Sex**				
Male	291 (82.90%)	38 (88.37%)	0.831	0.362
Female	60 (17.09%)	5 (11.63%)		
**Smoke**				
Yes	22 (6.27%)	3 (6.98%)	0.032	0.857
No	329 (93.73%)	40 (93.02%)		
**Drink**				
Yes	8 (2.28%)	2 (4.65%)	0.871	0.351
No	343 (97.72%)	41 (95.35%)		
**BMI(kg/m** ^**2**^ **)**				
< 28	330 (94.02%)	42 (97.67%)	0.972	0.324
≥ 28	21 (5.98%)	1 (2.33%)		
**Preoperative blood routine (white blood cell count)**				
Normal	226 (64.39%)	21 (48.84%)	3.960	0.047
Abnormal	125 (35.61%)	22 (51.16%)		
**With diabetes**				
Yes	22 (6.27%)	2 (4.65%)	0.006	0.936
No	329 (93.73%)	41 (95.35%)		
**Anatomical classification**				
Perianal subcutaneous	253 (72.08%)	23 (53.49%)	9.750	0.045
Pelvis	9 (2.56%)	3 (6.98%)		
Submucosal	5 (1.42%)	2 (4.65%)		
Deep retrorectal abscess	18 (5.13%)	5 (11.63%)		
Ischium	66 (18.80%)	10 (23.26%)		
**Abscess space**				
Low bit	319 (90.89%)	33 (76.74%)	6.625	0.010
High bit	32 (9.11%)	10 (23.26%)		
**Preoperative antibiotic use**				
Yes	51	8	0.500	0.480
No	300	35		
**Horseshoe abscess**				
Yes	76	10	0.058	0.810
No	275	33		
**Onset time**	8.79 ± 8.76	8.40 ± 7.55	t = 0.286	0.775
**Preoperative MRI**				
Yes	159 (45.30%)	12 (27.90%)	4.717	0.030
No	192 (54.70%)	31 (72.10%)		
**Surgical method**				
Hanging line drainage	251 (71.51%)	21 (48.84%)	9.212	0.002
Simple drainage	100 (28.49%)	22 (51.16%)		

MRI: magnetic resonance imaging; BMI: body mass index

There were statistically significant differences between the two groups regarding whether the patient received a preoperative MRI examination, the surgical method, the preoperative blood routine, the range of abscess cavity space and the anatomical classification (*p* = 0.030, *p* = 0.002, *p* = 0.047, *p* = 0.010 and *p* = 0.045, respectively). The results showed that the abnormal rate of preoperative blood routine results in the uncured group (51.16%) was higher than in the cured group (35.61%); the rate of perianal subcutaneous in the cured group (72.08%) was higher than in the uncured group (53.49%); the rate of high abscess space in the uncured group (23.26%) was higher than in the cured group (9.11%); The proportion of patients in the uncured group who underwent MRI before surgery (27.90%) was lower than in the cured group (45.30%); the proportion of patients in the uncured group who underwent simple drainage (51.16%) was higher than in the cured group (28.49%).

### Multivariate logistic regression analysis of prognosis of perianal abscess after operation

Based on the results of univariate analysis, multivariate logistic regression analysis was used to further determine the risk factors affecting the prognosis of perianal abscess after surgery. Whether the perianal abscess is cured after the operation is the dependent variable, and the routine blood range before the operation, MRI examination of the perineum, the range of the abscess cavity, and the operation method are the independent variables, which are included in the logistic regression analysis. The assignment is shown in Table 2. The results are shown in Table 3, the range of the abscess space (OR = 2.544, 95%CI = 1.087–5.954, *p* = 0.031) and the operation method (OR = 2.180, 95%CI = 1.091–4.357, *p* = 0.027) are independent influencing factors for the postoperative cure of perianal abscess.

**Table 2:** Assignment situation

Factor	Assignment
Preoperative blood routine range	Normal = 0; Abnormal = 1
MRI of the perineum before surgery	No = 0; Yes = 1
Abdominal space range	Low bit = 0; High bit = 1
Surgical approach	Simple = 0; Hanging line = 1

MRI: magnetic resonance imaging

**Table 3:** Logistic regression analysis of prognosis of perianal abscess after operation

Factor	B	SE	Wals	*P*	OR	95%CI
Preoperative blood routine range	0.494	0.346	2.037	0.154	1.639	0.831 − 3.233
MRI of the perineum before surgery	−0.520	0.386	1.812	0.178	0.595	0.279 − 1.267
Abdominal space range	0.934	0.434	4.633	0.031	2.544	1.087 − 5.954
Surgical approach	0.779	0.353	4.869	0.027	2.180	1.091 − 4.357

MRI: magnetic resonance imaging

### Discussion

The occurrence of anal fistula is more common in men than women, most often developing in the age range of 20–50, consistent with Amato A. et al. [23]. Compared with women, men have more unhealthy habits such as smoking, excessive drinking, staying up late, eating spicy food, etc., which leads to weakened immunity, intestinal dysfunction, acute and chronic enteritis, inflammatory bowel disease and other diseases that are more likely to cause anal glands infection [24–26]. Patients with perianal abscesses with long-term chronic systemic diseases are clinically more common with diabetes, and poor blood sugar control is a risk factor for long-term non-healing of wounds after perianal abscess surgery [27]. In this study, the univariate analysis of gender, age, and diabetes mellitus had no significant difference in the effect of postoperative abscess healing [28]. After patients with perianal abscesses are hospitalised, our hospital has repeatedly carried out rigorous health education for a long time to avoid unhealthy living and eating habits and long-term follow-up supervision during postoperative dressing changes; for patients with diabetes, postoperative blood glucose control is highly emphasised, thereby reducing the adverse effects of gender, age, diabetes and other factors in patients with perianal abscess surgery.

Perianal abscess is a common perianal disease. The treatment focus is improving the cure rate and reducing recurrence or the formation of anal fistula. It is crucial to evaluate the condition of the perianal abscess before surgery. The preoperative blood routine examination is a routine clinical examination. Although the specificity and sensitivity are poor, the low perianal abscess can often be manifested as a normal peripheral blood routine in this study, especially the subcutaneous abscess around the anus; the routine blood white blood cell count of patients with high perianal abscess is often high. Univariate analysis showed a statistically significant difference between white blood cell count and postoperative recurrence; multivariate analysis reveals it to be a non-independent influencing factor. Zhang Yingyi et al. identified that the mean platelet volume might thus be an indicator of perianal abscess severity [29]. In terms of device inspection, there have been significant advancements in clinical practice in recent years. In the past, only experienced clinicians used preoperative digital anal examinations to understand the extent and depth of the lesion, which caused considerable misjudgment. Intraoperative drainage, especially with high septal abscesses, is a risk factor for the postoperative cure of perianal abscesses. Advancements have been made in medical imaging, including the development of perianal rectal ultrasound, spiral computed tomography (CT), and MRI of the perineum. Despite these advancements, the clinical use of perianal rectal ultrasonography is limited, partly due to the local pain it may cause during the procedure and the requirement for clinicians to have experience in using B-mode ultrasound. Magnetic resonance imaging has been widely used in clinical treatment, especially high-resolution MRI, which can clearly show the extent of the lesion, the interval of the abscess cavity, and the internal mouth better than CT examination [23]. In this study, a preoperative MRI examination was performed. During the operation, the internal port was successfully found, and the thread-attached drainage accounted for 87.1%, which was significantly higher than that without the MRI examination, and the internal port-attached thread was 55.2%. The logistic regression analysis of intraoperative thread-hanging radical drainage and drainage is an independent influencing factor. It can treat the infected internal anal gland of abscess from the root cause and is better for postoperative curing than simple incision and drainage.

The data of this study has certain limitations. First, because it was a retrospective study, some patients’ contact information was missing; second, because of the acute onset of perianal abscess, some patients were treated from other hospitals and then admitted to our hospital for surgery, and the clinical data of the patients’ preoperative examinations were missing. As a result, data collection has a certain loss rate, which may impact data analysis. The sample research can be further expanded in the follow-up, and a higher level of evidence support can be provided through more complete clinical data.

In summary, the extent of the abscess cavity and the surgical method are independent factors for the recurrence of perianal abscess or the formation of anal fistula; the extent of blood routine and MRI examination are the risk factors for postoperative healing. Therefore, the severity of inflammation and infection can be indicated according to the range of blood routine before surgery; if the blood routine value is high in white blood cells, the MRI examination should be improved as much as possible. For deep and large-scale abscesses, the internal opening should be explored as much as possible during the operation, and adequate drainage should be made. For patients with chronic systemic diseases, appropriate active treatment can minimise the impact on the prognosis of patients with abscesses.

## Conclusion

Analysing the prognostic factors of perianal abscess surgery showed that the extent of abscess space and the surgical method were independent factors affecting the healing of postoperative perianal abscess, and the range of blood routine and perineal MRI were the risk factors affecting postoperative recovery. The preoperative assessment of the extent of the abscess cavity and the surgical method of abscess drainage significantly affect the prognosis of patients. In the future, it is worth discussing how to further evaluate the accuracy of the scope of the abscess cavity and clarify the relationship between the abscess cavity and the anal muscle space. Regarding the surgical method of abscess drainage, additional considerations should include the drainage materials and methods, how to accurately locate the internal opening during surgery and best practices for addressing it. The use of the intraoral approach, in particular, is worthy of further study.

## Data Availability

All data generated or analyzed during this study are included in this published article.
